# Transcription factor network downstream of protease activated receptors (PARs) modulating mouse bladder inflammation

**DOI:** 10.1186/1471-2172-8-17

**Published:** 2007-08-17

**Authors:** Ricardo Saban, Cindy Simpson, Carole A Davis, Igor Dozmorov, Julie Maier, Ben Fowler, Michael A Ihnat, Robert E Hurst, Barry K Wershil, Marcia R Saban

**Affiliations:** 1 Department of Physiology, The University Oklahoma Health Sciences Center, Oklahoma City, OK 73104, USA; 2 Oklahoma Medical Research Foundation (OMRF), Imaging Core Facility, Oklahoma City, Oklahoma 73104, USA; 3 Oklahoma Medical Research Foundation (OMRF), Arthritis and Immunology Research Program, Microarray/Euk. Genomics Core Facility, Oklahoma City, Oklahoma 73104. USA; 4 Department of Cell Biology, The University Oklahoma Health Sciences Center, Oklahoma City, OK 73104, USA; 5 Department of Urology, The University Oklahoma Health Sciences Center, Oklahoma City, OK 73104, USA; 6 Albert Einstein College of Medicine Division of Pediatric GI and Nutrition The Children's Hospital at Montefiore Bronx, NY 10467, USA

## Abstract

**Background:**

All four PARs are present in the urinary bladder, and their expression is altered during inflammation. In order to search for therapeutic targets other than the receptors themselves, we set forth to determine TFs downstream of PAR activation in the C57BL/6 urinary bladders.

**Methods:**

For this purpose, we used a protein/DNA combo array containing 345 different TF consensus sequences. Next, the TF selected was validated by EMSA and IHC. As mast cells seem to play a fundamental role in bladder inflammation, we determined whether c-kit receptor deficient (Kit^w^/Kit^w-v^) mice have an abrogated response to PAR stimulation. Finally, TFEB antibody was used for CHIP/Q-PCR assay and revealed up-regulation of genes known to be downstream of TFEB.

**Results:**

TFEB, a member of the MiTF family of basic helix-loop-helix leucine zipper, was the only TF commonly up-regulated by all PAR-APs. IHC results confirm a correlation between inflammation and TFEB expression in C57BL/6 mice. In contrast, Kit^w^/Kit^w-v ^mice did not exhibit inflammation in response to PAR activation. EMSA results confirmed the increased TFEB binding activity in C57BL/6 but not in Kit^w^/Kit^w-v ^mice.

**Conclusion:**

This is the first report describing the increased expression of TFEB in bladder inflammation in response to PAR activation. As TFEB belongs to a family of TFs essential for mast cell survival, our findings suggest that this molecule may influence the participation of mast cells in PAR-mediated inflammation and that targeting TFEB/MiTF activity may be a novel approach for the treatment of bladder inflammatory disorders.

## Background

Attesting to the importance of inflammation in disease, a trans-NIH Inflammation Working Group was formed recently to collect input on proposed research areas within the overarching theme of "Inflammation as a Common Mechanism of Disease" [1]. In general, inflammation plays a role in most bladder pathologies, including bladder cancer [2-5], and represents a defensive reaction to injury caused by physical damage, chemical substances, micro-organisms, or other agents [1,2]. In particular, several lines of evidence suggest that neurogenic bladder inflammation involves the participation of mast cells and sensory nerves. We previously demonstrated a key role for mast cells and their products in bladder inflammation [6-8]. As a consequence of inflammation, products of mast cell degranulation, such as tryptase, can be found in the urine of both bladder cancer and cystitis patients [9]. In addition to tryptase, other serine proteases, such as thrombin and trypsin, are produced during tissue damage and make important contributions to tissue responses to injury, repair, cell survival, inflammation [10-13], and pain [14-18]. Tissue responses to these enzymes are modulated by protease-activated receptors (PARs), a unique class of G protein-coupled receptors that use a fascinating mechanism to convert an extracellular proteolytic cleavage event into a trans-membrane signal. These receptors carry their own ligands, which remain cryptic until unmasked by receptor cleavage (for a review, please see references [14,17,19,20]).

Four PARs have been cloned so far, and all are co-expressed in the mouse bladder urothelium [21] and human urothelial cells [22]. In addition to the urothelium, PAR1 and PAR2 are also expressed in mouse detrusor muscle, and PAR4 is expressed in mouse peripheral nerves and plexus cell bodies [21,22]. The expression of PARs is altered during inflammation [21]. Additional evidence for the participation of PARs in the bladder inflammatory response was the finding that known pro-inflammatory stimuli, such as LPS, substance P, and antigen challenge, induce an increase in PAR4 RNA within hours of stimulation [23]. Moreover, up-regulation of PAR protein levels have been shown to be part of rat bladder responses to cyclophosphamide [24].

PAR1- and to a lesser extent PAR2-deficient mice exhibit a reduced response to bladder inflammation induced by *E. coli* lipopolysaccharide (LPS), substance P, and antigen [22]. The latter indicate that these receptors are upstream of a cascade of events leading to bladder inflammation [22,25]. In addition, regardless of the pro-inflammatory stimuli, the urinary bladder inflammatory transcriptome includes a sub-set of genes that are dependent on PAR activation [26].

**Figure 1:**
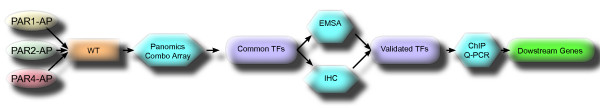
Molecular Approach. A top-down approach was used to determine transcription factors involved in the response of the mouse bladder to pro-inflammatory PAR-APs. This approach started by selecting a candidate TF out of 345 different TF consensus sequences present in a protein/DNA combo array (Panomics). Next, PAR-induced alteration in this TF binding was validated by EMSA and IHC was used to confirm its expression in the urinary bladder. Finally, an antibody recognizing this TF was used for CHIP/Q-PCR assay and revealed up-regulation of downstream genes.

Despite the evidence of PAR involvement in inflammation, pain, healing, and cancer in animal models, there seems to be no published clinical study regarding the efficacy of PAR antagonists. In order to search for therapeutic targets other than receptors themselves, we set forth to determine the transcription factors downstream of PAR activation as the next step in elucidating the network of responses at the molecular level to PAR activation. For this purpose, we introduced a new approach for the study of the transcriptional regulation downstream PAR activation (Figure 1). This top-down approach started by selecting a candidate TF out of 345 different TF consensus sequences present in a protein/DNA combo array (Panomics). Next, PAR-induced alteration in this TF binding was validated by EMSA and IHC was used to confirm its expression in the urinary bladder. Finally, an antibody recognizing this TF was used for CHIP/Q-PCR assay and revealed up-regulation of downstream genes.

## Methods

### Animals

All animal experimentation described here was performed in conformity with the "Guiding Principles for Research Involving Animals and Human Beings" (The studies have been reviewed and approved by the institutional review committee protocols 05-088I and 05-081). Genetically mast cell-deficient WBB6F1-Kit^w^/Kit^w-v ^and normal C57BL/6 mice were purchased from Jackson Laboratories (Bar Harbor, ME).

### Induction of cystitis

Acute cystitis was induced as we described previously [21,23,27-29]. Briefly, 8–10 week old female mice were anesthetized with isoflurane using a precision vaporizer, then transurethrally catheterized (24 Ga.; 3/4 in; Angiocath, Becton Dickson, Sandy, Utah), and the urine was drained by applying slight digital pressure to the lower abdomen. The urinary bladders were instilled on two consecutive days with 200 μl of one of the following substances: PAR-activating peptides (PAR1-AP = SFFLRN [30]; PAR2-AP = SLIGRL [30]; PAR4-AP = AYPGKF [31], and control inactive peptide = LRGILS [30]). All peptides were used at 10 μM concentration [22]. Substances were infused at a slow rate to avoid trauma and vesicoureteral reflux [18]. To ensure consistent contact of substances with the bladder, infusion was repeated twice within a 30-min interval and a 1-ml Tb syringe was maintained on the catheter end retaining the intravesical solution for at least 1 hour. Afterwards, the catheter was removed and the mice were allowed to void normally. Twenty-four hours post the second instillation, animals were euthanized by cervical dislocation and bladders were rapidly removed and frozen for immunohistochemistry or placed in cold PBS with protease (Protease Inhibitor Cocktail, Sigma, St. Louis, MO) and phosphatase inhibitors (20 mM sodium fluoride, 1 mM β-glycerophosphate, and 1 mM sodium orthovanadate) for separation of the urothelium/submucosa away from the detrusor smooth muscle.

### Separating the mucosa from the detrusor smooth muscle [32]

To decrease the complexity of using whole bladder homogenates, the bladder mucosa was separated from the detrusor smooth muscle, as described [32]. Briefly, immediately after removal from the animal, bladders were placed in PBS with protease inhibitors on ice and visualized under a dissecting microscope (Nikon SMZ 1500) and the detrusor smooth muscle was separated by blunt dissection away from the mucosa which contained the epithelium and sub-epithelial layers. Isolated layers were flash frozen and stored at -80°C until processing. Tissues were pulverized in a spring-loaded tissue pulverizer (Bio-Pulverizer, Biospec Products, Bartlesville, OK) and chilled with liquid nitrogen. Nuclear and cytosolic extracts were prepared using the Pierce NE-PER kit that enables stepwise separation and preparation of cytoplasmic and nuclear extracts from bladder tissue, as described [26]. Addition of the first two reagents (Pierce's proprietary information) to the pulverized tissue causes disruption of cell membranes and release of cytoplasmic contents. After recovering the intact nuclei from the cytoplasmic extract by centrifugation at 16,000 × g for 5 minutes, the nuclei are lysed with a third reagent (Pierce's proprietary information) to yield the nuclear extract. Extracts obtained with this product generally have less than 10% contamination between nuclear and cytoplasmic fractions – sufficient purity for most experiments involving nuclear extracts. A western blot was prepared using the nuclear and cytosolic extracts and probed for the nuclear proteins histone H3 and lamin A/C. No nuclear contamination was shown in the cytosolic fractions (data not shown). Protein concentrations were determined with a Micro BCA kit (Pierce, Rockford, IL) per manufacturer's instructions.

### Analysis of transcriptional regulatory network by protein/DNA combo arrays

Minimum Information About Microarray Experiments – MIAME [33].

#### Objective

To determine the transcriptional regulatory elements (TRE) downstream of the activation of PAR receptor. For this purpose, female C57BL6 mice (n = 3 per group) were instilled two times, 30 minutes part, with: control peptide, PAR1-AP, PAR2-AP, or PAR4-AP, every other day for 3 days. All peptides were used at 10 μM concentration. Mice were euthanized 24 hours after the last instillation and the urinary bladders were isolated, and the mucosa was separated from the detrusor muscle, as described above.

#### Array Design

Nuclear extracts from the bladder mucosa were obtained as detailed below for EMSA assays and analyzed by protein/DNA combo arrays containing 345 unique consensus-binding sequences per array [34].

#### Hybridization

To investigate the relative binding of TREs to their unique consensus sequences, we used the TransSignal protein/DNA combo array with spin column preparation (catalog no. MA1215) from Panomics (Fremont, CA) [34]. Array analysis was performed as per the manufacturer's instructions using nuclear extracts from mouse bladders before and after exposure to PAR-APs or control peptide. Nuclear extracts were prepared using the Panomics nuclear extraction kit (catalog no. AY2002) as per the manufacturer's instructions. Fifteen micrograms of nuclear extract were isolated before and after challenge, and they were incubated with 10 μl of TransSignal probe mix (Panomics) containing 345 biotin-labeled double-stranded DNA oligonucleotides. TFs bound to the double-stranded oligonucleotides were recovered by using the Panomics spin column. The biotin-labeled oligonucleotides specifically bound to the TREs were eluted and hybridized to the TransSignal array membrane containing oligonucleotides (representing 345 consensus binding sites for TREs) overnight at 42°C. The blots were then washed and incubated with a horseradish peroxidase (HRP)-conjugated streptavidin according to the manufacturer's instructions. The resulting spots were visualized on a FluorChem Imager (Alpha Innotech, San Leandro, CA). The images were quantified with AlphaEase FC software (Alpha Innotech) and the results were analyzed as described below.

#### Data normalization and analysis

We used the same methodology developed for analysis of cDNA microarrays [8,23,27,35,36]. Briefly, the raw data obtained with all Panomic membranes was first queried to determine only TFs expressed above background, as described for cDNA microarrays, and each expressed data was then log10 transformed. In addition, pairs of groups (control vs challenged) were adjusted to all data and then to each other using a robust regression analysis. Normalization was conducted using an iterative nonlinear curve-fitting procedure, as described [37]. This procedure assumes that intensities corresponding to TFs not expressed by the tissue will be normally distributed, and computes the mean and standard deviation (SD). Next, we normalized each expression profile to its own background (defined by adjusting a mean = 0 and SD = 1 of the distribution of non-expressed TFs). For further analysis, data obtained after normalization of each profile to its own background were log-transformed with substitution of negative values by the minimal logarithmic value obtained within positive values. Next, a robust regression analysis was conducted on expressed TFs. This analysis was based on the fact that, in a linear regression analysis between two compared samples, the majority of TFs are equally expressed and, therefore, randomly distributed around the regression line with a small portion of differentially expressed "outliers." The contribution of outliers to the regression analysis was down-weighted in an iterative manner. All expression profiles of both control and experimental groups were then rescaled to a common standard: the averaged profile of the control group. Our procedure for outlier exclusion was based on the selection of equally expressed TFs with close to normal distributed residuals (measured as deviations from the regression line). Only transcriptional factors exhibiting a ratio greater than 3.0 between control and treated groups are being presented.

#### Electrophoresis motility assays (EMSAs)

A commercially available double-stranded TFEB probe corresponding to the adenovirus major late promoter core sequence (5'-G TAG GCC ACG TGA CCG GG-3', 3'-C ATC CGG TGC ACT GGC CC-5', Santa Cruz Biotechnology, Santa Cruz, CA) was constructed by end-labeling a double-stranded probe with γ^32^P ATP (3000 Ci/mmole; GE Healthcare) and T4 polynucleotide kinase (New England Biolabs) and then purified using a G-50 column (GE Healthcare). A second probe corresponding to the μE3 immunoglobulin enhancer site (5'-GAT CTG GTC GTG TGG CAA GGC-3', 3'-CTA GAC CAG TAC ACC GTT CCG-5'; Santa Cruz Biotechnology) was also tested but it was found to have a much lower binding affinity as previously described by others [38] (data not shown).

Nuclear extracts (10 μg) from the bladder mucosa and detrusor muscle were placed in 10 mM Tris HCl pH 7.9, 100 mM KCl, 5 mM MgCl_2_, 1 mM EDTA, 5% glycerol, 0.02% NP-40, 1 μg poly dI-dC, 1 mM DTT with protease and phosphatase inhibitors were added to 10,000 cpm labeled TFEB consensus oligonucleotide and incubated at room temperature for 20 minutes. Cold reactions contained 100× unlabeled TFEB consensus oligonucleotide. For super-shift reactions, 10 μg of nuclear extract was pre-incubated for 30 minutes on ice with 1.5 μg TFEB antibody (Abcam, Cambridge, MA). Reactions were resolved on a 5% native acrylamide gel with 0.5 × TBE at 300 V for 2 hours. Gels were vacuum-dried and visualized on Kodak Biomax MS Film and quantified using ImageJ Software (NIH) [39].

#### TFEB Immunofluorescence

Frozen bladders were processed for routine immunohistochemistry according to published methods [27]. All reagent incubations and washes were performed at room temperature. Normal blocking serum (Jackson Immunolabs) was placed on all slides for 45 min and sections were incubated with primary antibody (Goat anti-TFEB N-term 2–14, ab2636 Abcam) at concentrations of 1, 2, and 4 μg/ml. Controls include omission of the primary antibody. Slides were washed and incubated with secondary antibodies (Alexa Fluor 546 donkey anti-goat IgG, A11056, Invitrogen). Slides were washed, counterstained with DAPI and coverslipped with Shur/Mount (TBS) mounting media. All tissues were imaged at room temperature using Axiovision 4.6 and a Zeiss axiocam HRm high-resolution CCD camera. Selected sections were visualized and photographed using a Zeiss LSM510 Laser Scanning Confocal with META (Thornwood, NY). TFEB fluorescence was analyzed in triplicate by Image analysis of the urothelial cell layer. Images were analyzed with Image-Pro Analyzer^® ^(Media Cybernetics Inc.; Silver Spring, MD) and the area expressing positive cells for TFEB at 200× magnification was calculated as percent of the total urothelial cell area. Results are presented as average and standard error of the mean.

#### Target validation by Q-PCR of Chromatin Immunoprecipitation (CHIP)-Based Assays

Target validation was sought for 7 genes known to have an E-box based on the evidence on the literature (E-cadherin, serpine 1, IGF1R, WT1, cyclin D1, and cathepsin K) or our own observation Eif4ebp2 [32]. Female C57BL/6J mice (n = 20 per group) were anesthetized (ketamine 200 mg/kg and xylazine 2.5 mg/kg, i.p.), and instilled on two consecutive days with 200 μl of one of the following substances: PAR2-AP [SLIGRL [30]] or control inactive peptide [LRGILS [30]]. Twenty-four hours after instillation, mice were euthanized with pentobarbital (200 mg/kg, i.p.) and the whole bladders removed rapidly and frozen.

Frozen bladders were shipped to Genpathway [40] for querying the chromatin for detection and quantification of TFEB binding upstream of specific genes using chromatin immunopreciptation (ChIP) combined with real time PCR (Q-PCR) (Genpathway's FactorPath Query assay) [41]. The whole bladders, contained both the mucosa and detrusor layers, were exposed briefly to formaldehyde for cross-linking of the proteins and DNA together, followed by sonication to fragment the DNA into pieces of approximately 300–500 bp. The same TFEB antibody used for IHC (Abcam, Cambridge, MA) was then used to precipitate the TFEB-DNA complexes. The Ab-protein-DNA complexes were purified using beads coupled to protein G. The DNA was isolated from the complexes using a combination of heat to reverse cross-linking, RNase and proteases, and then purified using phenol extraction and EtOH precipitation. The final ChIP DNAs were then used as templates for Q-PCR reactions using primer pairs specific for each genomic region of interest. Q-PCR was carried out using *Taq* polymerase (iQ SYBR Green Supermix, Bio-Rad). Primer pairs were designed using Primer 3 [42]. Details of the primer sequences and the Genebank accession numbers are given in Table 1. The designed primers shared 100% homology with the target sequence but no significant homology with other sequences.

**Table 1:** PRIMERS FOR CHIP/Q-PCR

SYMBOL	DESCRIPTION	MOUSE REF	FORWARD PRIMER	REVERSE PRIMER
Cyclin D1	Cyclin D1	NM_007631	agagcttagggctcgtctgg	agcgtccctgtcttctttca
Ctsk	Cathepsin K	NM_007802	atgttgaggggacagaggtg	gcttctgggcatggagtagt
Eif4ebp2	eukaryotic translation initiation factor 4E	NM_010124	gctccacccttcaacacttc	gggagggacaatatggaagc
Wt1	Wilms tumor homolog	NM_144783	gccagagaggagggtgtctc	ATTCACACAgcagccctagc
Serpine1	serine (or cysteine) proteinase inhibitor, clade	NM_008871	ttccggctcacatctggtat	ATTGGCTCTTGTTGGCTGTC
Igf1r	insulin-like growth factor I receptor	NM_010513	aaagtgccttgcgtagcagt	tttcgcagtgtggtggaaag
E-Cadherin	cadherin 1	NM_009864	tcgggagactgaaacaggag	agagggtcttgggattgcat

Q-PCRs reactions were run in triplicate and the averaged Ct values were transferred into copy numbers of DNA using a standard curve of genomic DNA with known copy numbers. The resulting TFEB binding values for each genomic region were also normalized for primer pair amplification efficiency using the Q-PCR values obtained with Input DNA (unprecipitated genomic DNA). Results are presented as binding events detected per 1000 cells for each genomic region tested and compared to an untranscribed region used as a negative control. The difference between two mean values was analyzed with an unpaired Student's *t*-test (GraphPad Prism software version 4.0; GraphPad Software, Inc. San Diego, CA). A nominal *p *value less than 0.05 was considered statistically significant.

#### Materials

PAR1-AP, PAR2-AP, and PAR4-AP and control peptide were synthesized at the Molecular Biology Resource Facility, William K. Warren Medical Research Institute, OUHSC, as carboxyl-terminal amides, purified by high-pressure liquid chromatography, and characterized by mass spectroscopy. Peptide solutions were made fresh in PBS from powder. PAR3-AP was not used in this research because of lack of specificity.

## Results

### Protein/DNA combo array

**Figure 2:**
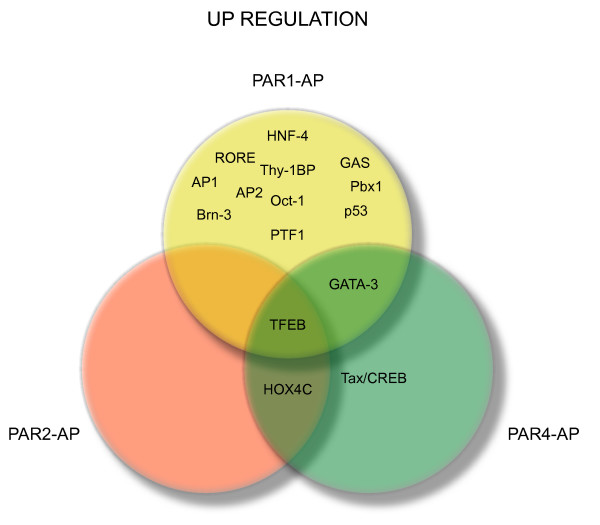
**Up-regulated TFs downstream of PAR activation**. C57BL/6 mice were instilled on two consecutive days, with 200 μl of a 10 μM solution of the PAR1-AP, PAR2-AP, PAR4-AP, or control peptide. Twenty four hours after instillation, the bladders were removed, placed on ice with peptidase inhibitors, and the urothelium/submucosa was isolated. Nuclear extracts from the bladder mucosa were analyzed by protein/DNA combo arrays containing 345 unique consensus-binding sequences per array [34]. TF expression was normalized to its own background (defined by adjusting a mean = 0 and SD = 1 of the distribution of non-expressed TFs). For further analysis, data obtained after normalization of each profile to its own background were log-transformed with substitution of negative values by the minimal logarithmic value obtained within positive values. Next, a robust regression analysis was conducted on expressed TFs and only TF with a ratio > 3.0 between PAR and control are represented.

**Figure 3:**
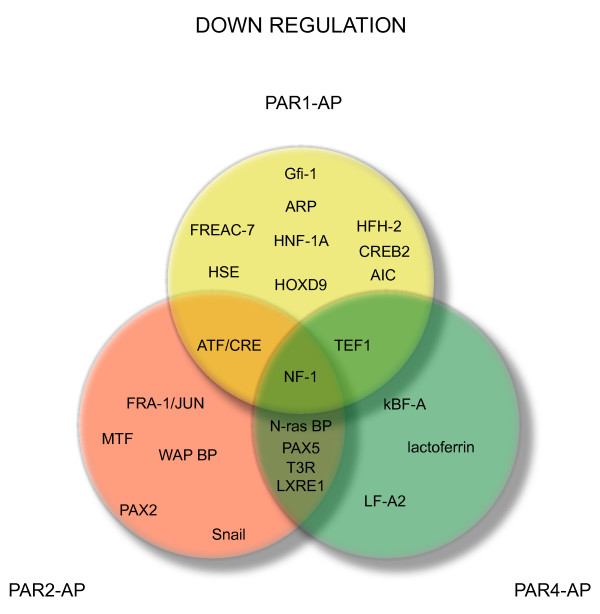
**Down-regulated TFs downstream of PAR activation**. Down-regulated TFs were calculated as described in Figure 2 and only TF with a ratio < 3.0 between PAR and control peptide are represented.

**Figure 4:**
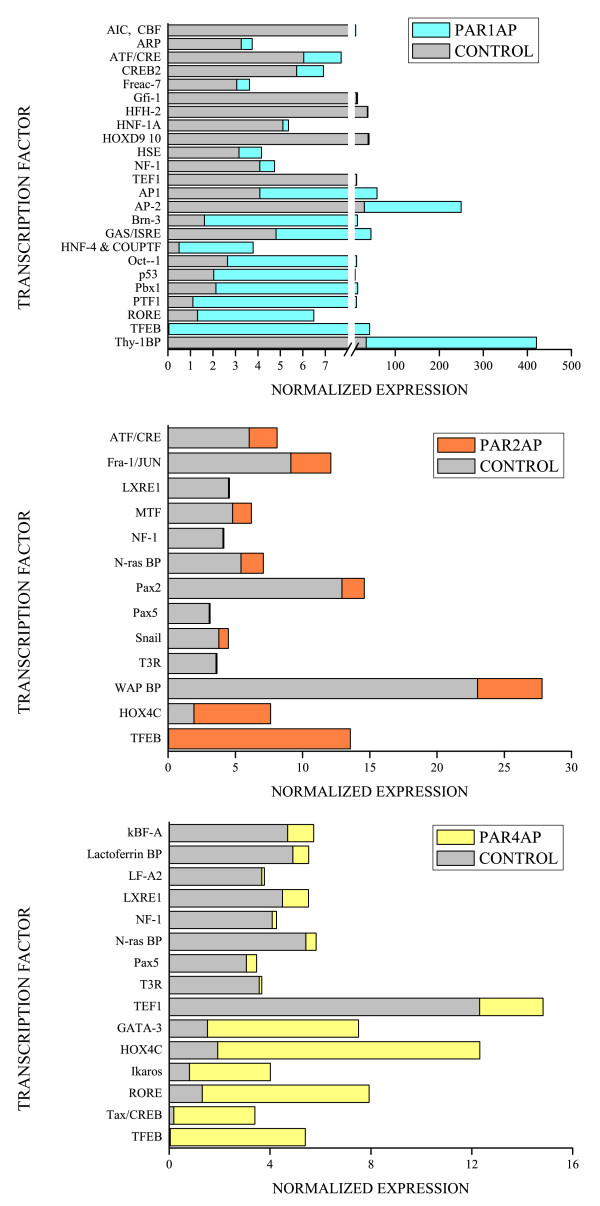
**Relative expression of TFs in response to PAR1, PAR2, and PAR4 activation**. Both up- and down-regulated transcription factors altered by a factor greater than 3 are represented. Listed TFs are summarized on Table 2.

PAR1-activating peptide (AP), PAR2-AP, PAR4-AP, and control peptide were instilled into the mouse bladder at the concentration of 10 μM. This treatment induces a strong and reproducible bladder inflammation, whereas no inflammation was observed in mice instilled with 10 μM control peptide [22]. We used a protein/DNA combo array that separated bound DNA/protein complexes from unbound probe. The biotin-labeled probe/protein was hybridized to a membrane containing 345 transcription factor consensus sequences. As this assay closely resembles the membrane cDNA arrays, we used the same methodology developed by our collaborator (ID) to analyze the results [27,35,43]. Figure 2 illustrates the TFs that were at least 3-fold up-regulated and Figure 3 represents those TFs that were 3-fold down-regulated by each PAR-AP. Table 2 summarizes all TFs. The relative magnitude of the different TF signals between PAR-APs is illustrated in Figure 4. These results strongly suggested that TFEB, a member of the MiTF family of bHLH-Zip transcription factors, was the only TF commonly altered by PAR1-AP, PAR2-AP, and PAR4-AP and therefore, occupied the center of Venn diagram (Figure 2).

**Table 2:** Transcriptional Factors altered by PAR-AP stimulation of the mouse bladder mucosa as detected by Protein/DNA combo array.

**Upregulated**	
AP-1	Fos, FosB, Fra1, Fra2, Jun, JunB [82]
AP-2	Activator protein 2 [83]
Brn-3	POU4F1: POU domain, class 4, transcription factor 1 [84]
GAS/ISRE	Gamma-interferon activation site [85]
GATA-3	GATA binding protein3 (globin transcription factor 3) [86]
HNF-4	Hepatocyte nuclear factor 4 [87]
HOX4C	homeo box4C [88]
Oct-1	POU2F1: POU domain, class 2, transcription factor 1 [89]
p53	TP53: tumor protein p53 [90]
PBX1	pre-B-cell leukemia transcription factor 1 [91]
PTF1	pancreas specific transcription factor [92]
RORE	RAR-related orphan receptor [93]
Tax/CREB	TAX & CREB complex-responsive protein [94]
TFEB	TFEB is a the b-HLH-LZ closely related to TFE3 [38]
Thy-1 BP	Thy-1binding protein [38]

**Down Regulated**	

AIC, CBF	Apolipoprotein AI (ApoAI) promoter c region, CCAAT-binding factor ([95]
ARP	COUP-beta; apolipoprotein AI; NR2F2 ([95]
ATF/CRE	Activating transcription factor [96]
CREB2	cyclic AMP response element binding protein 2 [97]
Fra-1/JUN	Fos-related antigen [98]
Freac-7	Forkhead box L1 [99]
Gfi-1	Growth factor independent 1 [100]
HFH-2	Forkhead box D3 [101]
HNF-1A	Hepatocyte nuclear factor 1 [102]
HOXD9	HOXD9,10 [88]
HSE	Heat shock transcription factor [103]
kBF-A	kappa immunoglobulin enhancer binding protein [104]
Lactoferrin BP	Lactoferrin enhancer binding region for estrogen receptor [105]
LF-A2	Liver-specific transcription factor [106]
LXRE1	Nuclear receptor subfamily 1, group H, member 2 [107]
MTF	MRE-binding transcription factor-1 [108]
NF-1	CTF; NF-I; TGGCA-binding protein [109]
Pax2	Pax-2 DNA-binding transcription factor [110]
Pax5	Pax-5 DNA-binding transcription factor [111]
Snail	Zinc-finger transcription factor Snail [112]
T3R	c-ErbA; thyroid hormone receptor [113]
TEF1	TEF 1 box [114]
WAPBP	Whey acidic protein binding protein [115]

### Immunohistochemistry

**Figure 5:**
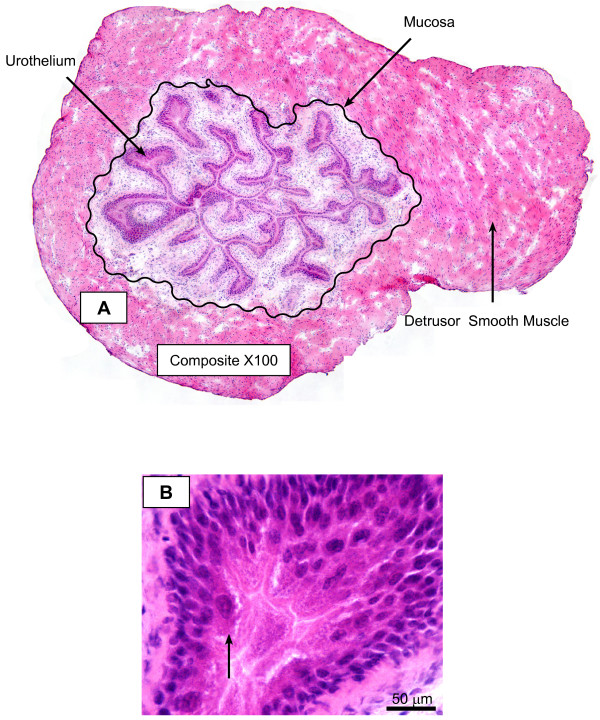
Representative photomicrograph of the expected morphology of the urinary bladder in a cross-section. Figure 5A is a composite of 18 H&E stained photomicrographs of the same cross-section at original magnification of 100× and illustrates the localization of the different layers: urothelium and submucosa, as well as the detrusor muscle. Black wavy line indicates the urothelium/submucosal layer. Figure 5B is a high magnification of the mouse bladder urothelium showing a multi-layered urothelium with apical umbrella cells (yellow arrow).

**Figure 6:**
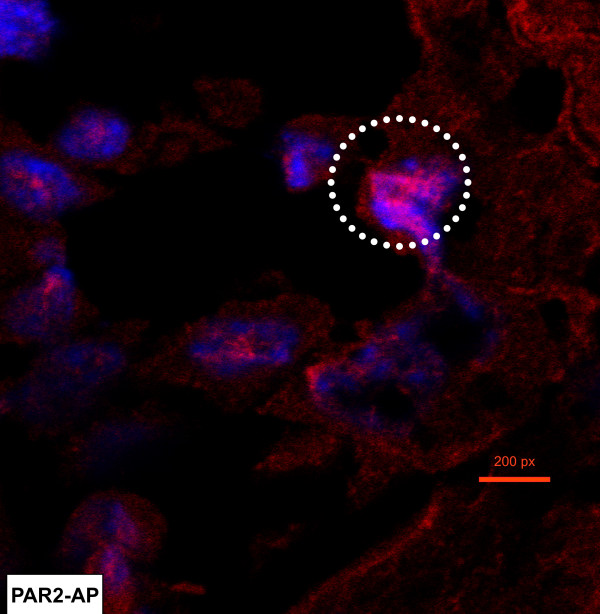
Representative photomicrograph of the bladder urothelium isolated from a C57BL/6 mouse challenged with PAR2-AP (10 μM). White circle indicates the expression of TFEB, particularly in the nucleus (DAPI-positive) of the urothelial cells.

**Figure 7:**
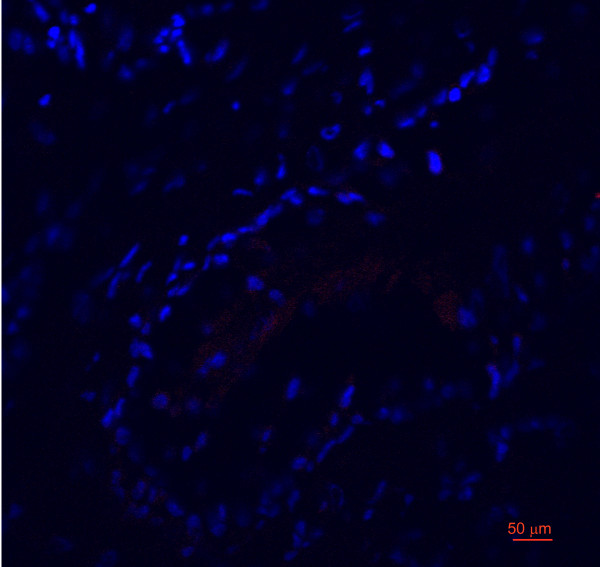
Representative photomicrograph of the mouse bladder urothelium isolated and stained with DAPI and secondary antibody.

**Figure 8:**
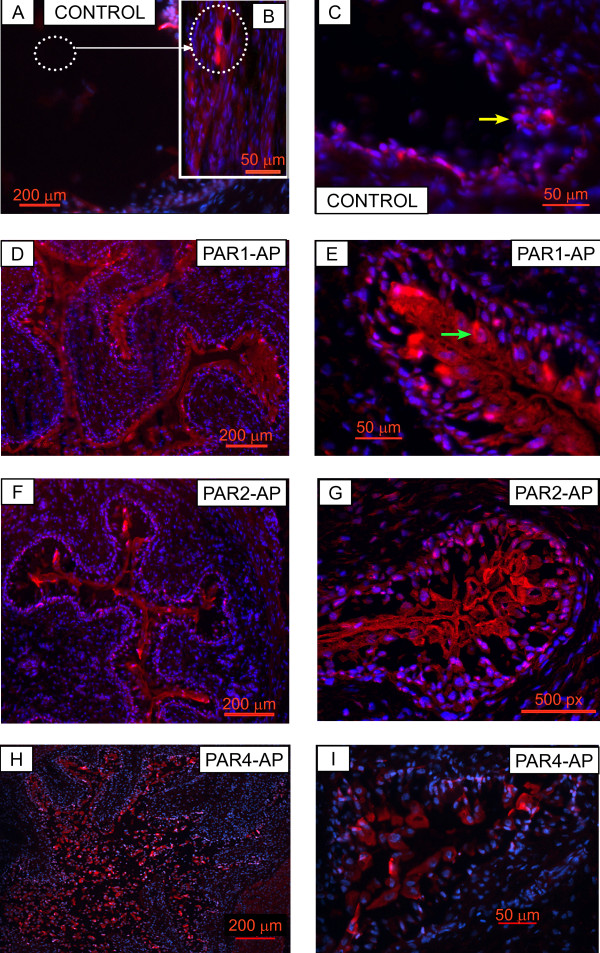
Representative photomicrographs illustrating a constitutive TFEB expression in the urothelium (8 A-C) and smooth muscle 8A of C57BL/6 mice stimulated with control peptide. The insert, Figure 8B is a high magnification of the area highlighted in Figure 8A. Figures 8D and 8E are representative images of tissues isolated from PAR1-AP-treated C57BL/6 mice and illustrate an overwhelming TFEB expression throughout the urothelial layer, including in umbrella cells (green arrow, Figure 8E). Figures 8 F-G and 8 H-I are representative of responses to PAR2-AP and PAR4-AP, respectively. Yellow arrow indicates the urothelium (8C) and dotted white circle indicate TFEB positive cells in the detrusor smooth muscle (Figures 8A and 8B).

**Figure 9:**
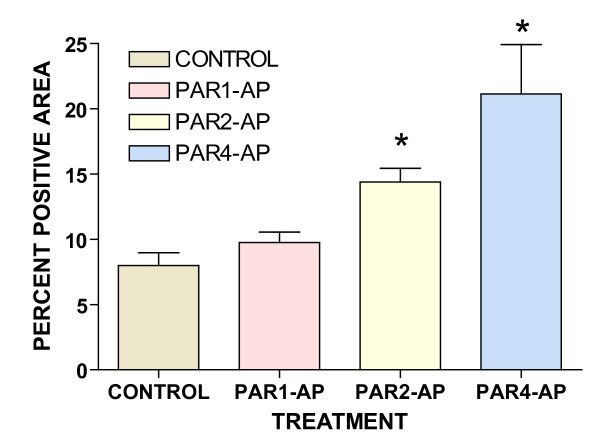
Quantification of 3 replications of TFEB Immunohistochemistry. Asterisks indicate a statistically significant difference (p < 0.05) between PAR-APs and control peptide in inducing TFEB immuno stain.

To confirm whether the urinary bladder expresses TFEB, we used a commercially available antibody to localize its expression. Figure 5A–B represents the expected morphology of the urinary bladder in a cross-section. Figure 5A is a composite of 18 H&E stained photomicrographs of the same cross-section at low magnification (×100), and illustrates the localization of the different layers: urothelium and submucosa, as well as the detrusor muscle. Figure 5B is a high magnification of the mouse bladder urothelium showing a multi-layered urothelium with apical umbrella cells (arrow). Figure 6 is a representative image of a bladder isolated from a mouse challenged with PAR2-AP (10 μM), which indicates the expression of TFEB, particularly in the nucleus (DAPI-positive) of the urothelial cells. Figure 7 is a representative photomicrograph of the mouse bladder stained with secondary antibody. Figures 8A–I are representative photomicrographs illustrating TFEB expression in the bladder cross sections. Figures 8A–C show the constitutive expression of TFEB in the urinary bladders isolated from mice stimulated with the control peptide, including the urothelium (Figures 8A–C) and detrusor muscle. A higher magnification insert depicts the expression in the smooth muscle (Figure 8B). Figures 8D and 8E are representative images of tissues isolated from PAR1-AP-treated mice and illustrate an overwhelming TFEB expression throughout the urothelial layer, including umbrella cells (Figure 8E). Figures 8F–G and 8H–I are representative of responses to PAR2-AP and PAR4-AP, respectively. Overall, our results indicate a constitutive, although low, expression of TFEB in the control bladder urothelium. Bladder instillation with PAR-APs induced a substantial induction of TFEB expression, primarily in the urothelium, that was clearly observed in response to PAR2-AP and PAR4-AP, and to a lesser extent, with PAR1-AP. Figure 9 presents the quantification of 3 replications of TFEB immunohistochemistry.

### Absence of PAR-induced bladder inflammation in *c-kit*-deficient mice

**Figure 10:**
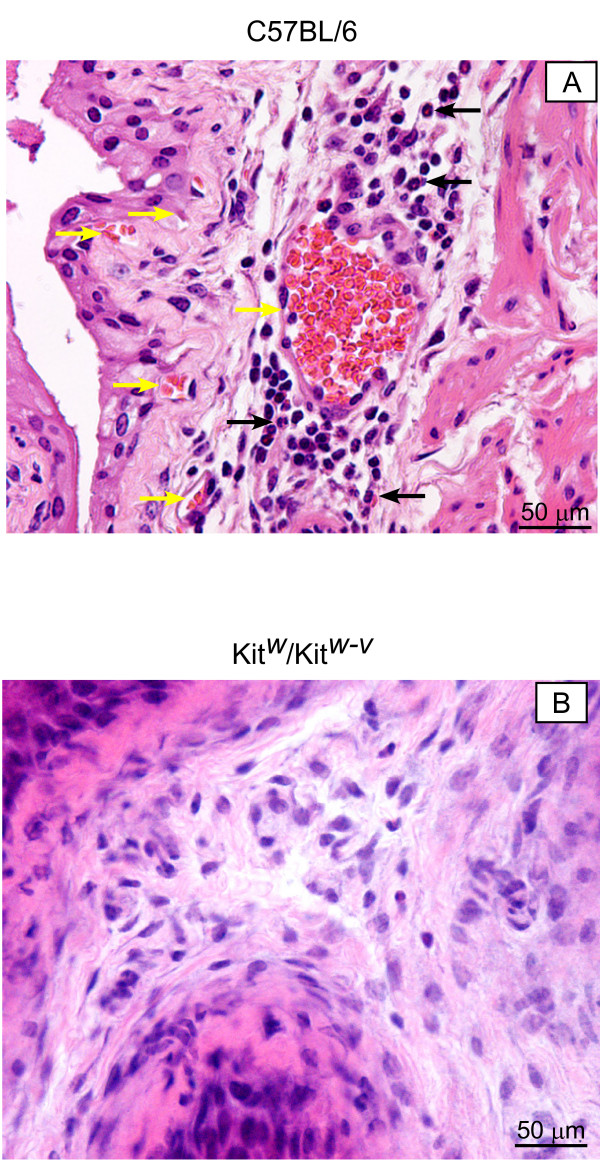
Comparison of bladder inflammation in response to 10 μM of PAR1-AP in wild type (C57BL/6) and c-kit receptor deficient (Kit^w^/Kit^w-v^) mice. A = Representative photomicrographs of H&E stained C57BL/6 urinary bladders following PAR1-AP (10 μM)-induced inflammation. PAR1-induced inflammation was characterized in C57BL/6 mice by an intense vasodilation (yellow arrows) and polymorphonuclear leukocyte infiltration (black arrows). B = absence of bladder inflammation in bladders isolated from Kit^w^/Kit^w-v ^mice challenged with PAR1-AP.

Direct evidence for an essential role for mast cells in cystitis was reported by our laboratory in animal models of bladder inflammation [7,8,44]. However, the responses to PAR-APs were not investigated. Therefore, we compared the bladder inflammatory responses to PAR1-AP in wild type and Kit^w^/Kit^w-v ^mice. As shown in Figure 10, PAR1-AP induces inflammation in wild type mice, consistent with our previous published results [21,22]. This inflammation was characterized by intense inflammatory infiltrate in the submucosa and vasodilation (Figure 10A). In contrast, Kit^w^/Kit^w-v ^did not develop an inflammatory response to PAR1 activation (Figure 10B).

### Electrophoretic mobility shift assay (EMSA)

**Figure 11:**
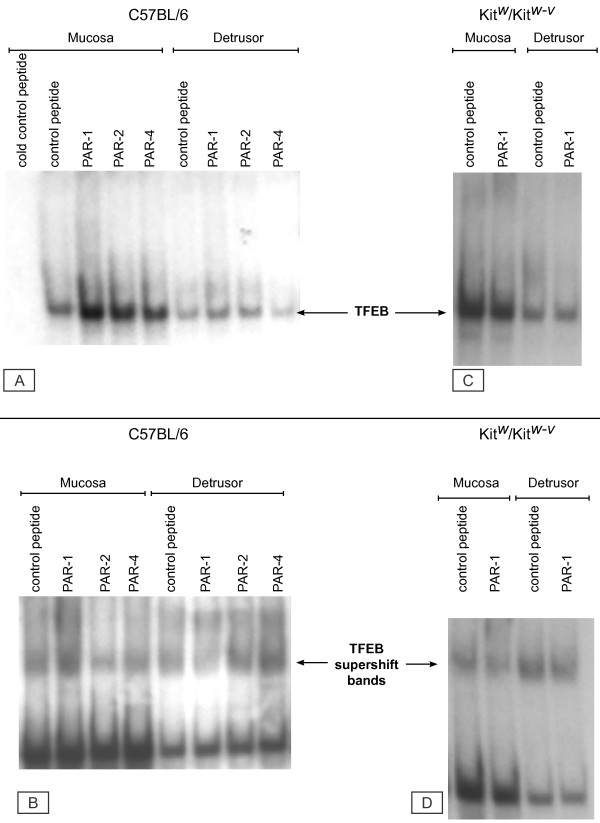
**Electrophoretic mobility shift (EMSA) and gel shift assays**. A) EMSA was performed with C57BL/6 mice treated intravesically with control peptide, PAR1-AP, PAR2-AP, or PAR4-AP, at a concentration of 10 μM. Following treatment, the bladders were removed and the urothelium/submucosa was separated from the detrusor smooth muscle, and nuclear extracts were obtained from both layers. Shifted bands were observed with all treatments. C) No significant increase was observed in mast cell deficient Kit^w^/Kit^w-v ^mice challenged with PAR1-AP. For super-shift assays (B and D), reactions were incubated for 30 minutes at 4°C with TFEB antibody, prior to addition of the probe.

**Figure 12:**
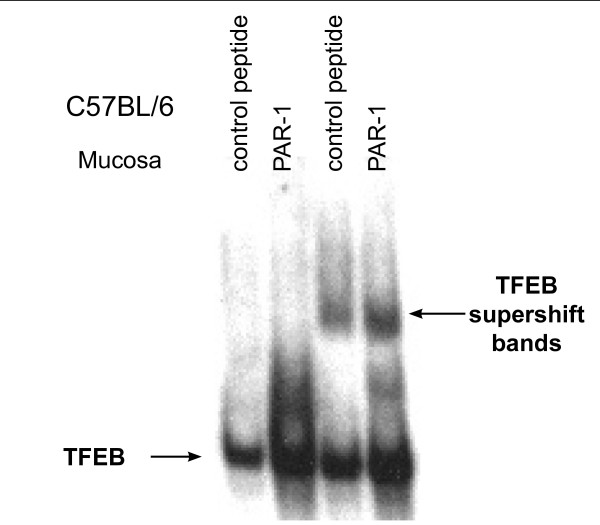
Additional TFEB EMSA performed in tissues isolated from C57BL/6 mice that were challenged with the control peptide and PAR1-AP (10 μM).

**Figure 13:**
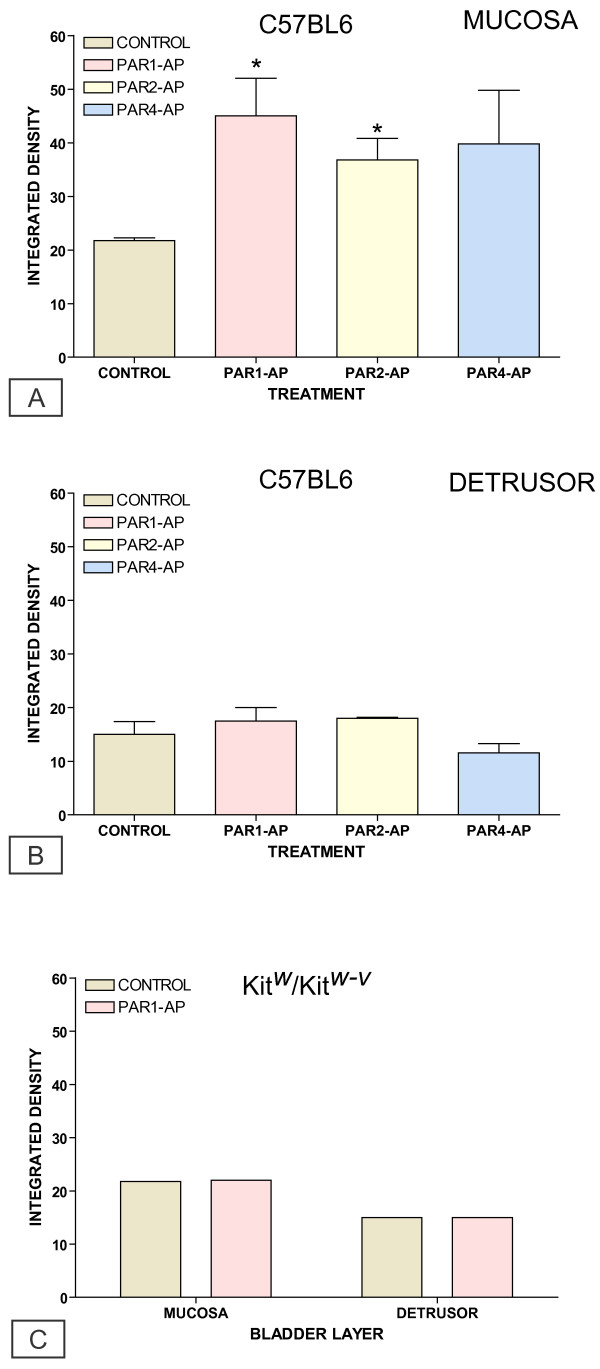
**Quantification of EMSA results**. Graphical representation of the integrated density for TEFB EMSAs in bladder mucosa (A) and detrusor muscle (B) isolated from C57BL/6 mice treated with the control peptide, PAR1-AP, PAR2-AP, and PAR4-AP, and tissues isolated from kit^w^/kit^w-v ^mice challenged the control peptide and PAR1-AP (C). Asterisks indicate a statistically significant difference (p < 0.05) between PAR-APs and control peptide in inducing an increase in TFEB binding activity.

We used the same binding sequence present in the Panomics array [34], as the TFEB EMSA probe. Our results indicate that intravesical instillation of C57BL/6 mice with all PAR-APs increased the binding activity to the TFEB probe primarily in extracts of the bladder mucosa (Figure 11A). In contrast, no significant increase was observed in mast cell deficient Kit^w^/Kit^w-v ^mice challenged with PAR1-AP (Figure 11C). In addition to TFEB, other members of the microphthalmia (MiTF)-TFE subfamily of basic helix-loop-helix leucine zipper (bHLH-ZIP) transcription factors are known to bind to this E-Box sequence [45-47]. Therefore, we used a TFEB antibody to reveal a specific TFEB activity, as illustrated by the super shift bands in tissues isolated from C57BL/6 (Figure 11B) and Kit^w^/Kit^w-v ^mice (Figure 11D). Next, we determined the variability of the EMSA by performing additional experiments in tissues isolated from C57BL/6 mice that were challenged with the control peptide and PAR1-AP (10 μM), Figure 12. ImageJ software was used to quantify the EMSA signals and the integrated density is presented on Figures 13A–C

### Target validation by Q-PCR of Chromatin Immunoprecipitation (CHIP)-Based Assays

**Figure 14:**
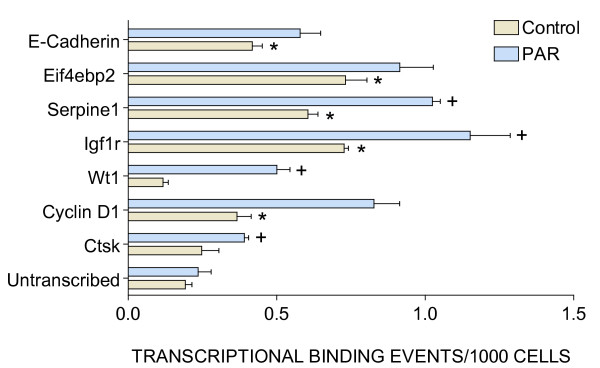
**Target validation by Q-PCR of Chromatin Immunoprecipitation (CHIP)-Based Assays**. Female C57BL/6J mice were anesthetized and instilled with 200 μl of control peptide or PAR2-AP on days 1 and 2, as described in methods. Mice were euthanized 24 hours after the last instillation; the urinary bladders were removed rapidly, frozen, and shipped to Genpathway [40] for querying the chromatin for transcription of selected genes. After isolation, the chromatin was incubated with TFEB antibody to precipitate the CHIP DNA. The final CHIP DNAs were then used as templates in Q-PCR reactions, performed in triplicate, using specific primer pairs (Table 1). Results are presented as average and standard error of Transcription Binding Events Detected Per 1000 Cells. Asterisks indicate a statistical significant difference (p < 0.05) between a specific gene and the un-transcribed region used as control and a plus sign indicates a statistical significant difference (p < 0.05) between CHIPs isolated from PAR2-AP- and control peptide-treated bladders.

In order to investigate whether the increased expression of TFEB would translate into an up-regulation of genes known to contain an E-box on their promoter, we used a combination of chromatin immunopreciptation (CHIP) and real-time polymerase chain (Q-PCR). For this purpose, we used the same TFEB antibody employed for IHC. CHIP was obtained from the whole bladder isolated from PAR2-AP- and control peptide-treated bladders. We queried the expression of selected genes known to have an E-box on their promoter and compared the results with an un-transcribed region of the DNA. Figure 14 indicates an increased number of events (p < 0.05) on the CHIP precipitated with TFEB antibody when compared to the un-transcribed region for the following genes: e-cadherin, eif4bp2, serpine 1, IGF1R, and cyclin D1. In addition, inflammation associated with PAR2 stimulation led to an up-regulation of serpine 1, WT1, cyclin D1, IGF1R, and cathepsin K.

## Discussion

We used a top-down approach for determination of transcription factors involved in the response of the mouse bladder to pro-inflammatory PAR-APs (Figure 1). First, a Protein/DNA combo array was used to select transcription factors common to all PAR activation. Out of 345 TFs present in this array, only TFEB was shared by PAR1-, PAR2-, and PAR4 activation. Next, we confirmed that TFEB is expressed in the mouse bladder by IHC. Finally, we compared the inflammatory responses to PAR1-AP between wild type and mast cell-deficient Kit^w^/Kit^w-v ^mice. Kit^w^/Kit^w-v ^mice failed to mount an inflammatory response to PAR1-AP, and EMSA confirmed that TFEB is upregulated by all PAR-APs in wild type mice but not in Kit^w^/Kit^w-v ^mice. Finally, we used a combination of chromatin immunoprecipitation and QPCR to target validate transcripts known to be under the control of TFEB. This assay takes into consideration genes that are actively transcribed, in contrast to cDNA array technologies that queries RNA accumulation. The disadvantage of the CHIP/Q-PCR method is the amount of chromatin necessary for CHIP, which limits this analysis to the whole bladder and not to specific layers (mucosa and detrusor) used for EMSA. Therefore, our CHIP/Q-PCR results have to be taken in the context that migrating inflammatory cells, in addition to resident cells, contributed to the measured values. These results indicate that TFEB is downstream of PAR activation, that this response occurs primarily in the bladder urothelium, and that TFEB mobilization depends on the presence of either mast cells or alternatively c-kit receptors, which are expressed on a variety of tissues, including certain nerves. Given our previous findings demonstrating the importance of mast cells in bladder inflammation and our finding of MiTF expression, we speculate that the mast cell is the key contributor to this response, but further studies will be necessary to delineate the precise mechanism of TFEB mobilization.

TFEB belongs to the microphthalmia (MiTF)-TFE subfamily of basic helix-loop-helix leucine zipper (bHLH-ZIP) transcription factors which includes four family members: TFEB, TFE3, TFEC, and MiTF. MiTF family members are required for the proper development of osteoclasts, melanocytes, retinal pigment epithelial cells, mast cells, and natural killer cells [48]. In addition, MiTF has been implicated in the regulation of tissue-specific gene expression in mast cells [49].

Each protein of the MiTF family forms homo- or heterodimers with other family members [50] and recognizes, in general, the same DNA sequences [51]. In particular, TFE3 and TFEB elicit their effects mainly through the binding to M-box (AGTCATGTGCT) and E-box motifs (CACGTG) [52].

### MiTF family in the urinary tract

The only other finding implicating this family in the urinary tract is their involvement in renal cell carcinoma. In this regard, TFE3 is involved in chromosomal translocations recurrent in renal cell carcinoma [53]. Translocations of the genes encoding the related transcription factors TFE3 and TFEB are almost exclusively associated with a rare juvenile subset of renal cell carcinoma and lead to over-expression of TFE3 or TFEB protein sequences. TFE3 and TFEB have been identified as cell type-specific leukemia inhibitory factor-responsive activators of E-cadherin [54].

### MiTF and Mast Cells

MiTF mRNA and protein levels are higher in human mast cells than monocytes and granulocytes [55]. MiTF seems to be involved in the migration, phenotypic expression, and survival of mast cells [56]. As an essential transcriptional effector of the c-kit pathway, MiTF is critical for mast cell [49,56] and melanocyte development [49,57-59]. Mice deficient in MiTF harbor a severe mast cell deficiency, and MiTF-mutant mast cells cultured *ex vivo *display a number of functional defects [60]. In addition, *c-kit*- and m-Csf-dependent MAPK phosphorylation transcriptionally activates both TFE3 and MiTF, which is necessary for mast cell developmental functions [54]. Our findings indicate that the bladder inflammatory responses mediated by PAR1 are dependent on the presence of mast cells by a mechanism that involves TFEB. The latter adds to a list of evidence published by this laboratory indicating that Kit^w^/Kit^w-v ^mice do not develop bladder inflammation in response to antigen challenge [7], and substance P [7] stimulation, whereas reconstitution Kit^w^/Kit^w-v ^with mast cells from wild type mice restores the inflammatory response [7,8]. Therefore, we did not test whether the responses mediated by PAR2 and PAR4 are also dependent on the presence of mast cells.

### PARs and MiTF

Thrombin and tryptase are endogenous agonists of PARs in humans. Along with serine proteases, it seems that tissue factor and element of the coagulation cascade are also endogenous activators of PARs [61,62]. In human vascular endothelial cells both thrombin and PAR1-AP elicited gene regulation via E-box signaling [62]. However, the information regarding endogenous activators of PARs in the mouse bladder is, unfortunately, scanty. While human connective tissue mast cells contain the enzymes chymase and tryptase, mice contain numerous related proteases [63-65]. Mouse mast cell protease-7 is a tryptase predominantly expressed in differentiated connective tissue-type mast cells [66]. Mast cell proteases mcpt-5 (chymase), and the tryptases mcpt-6 and mcpt-7 are all expressed during the development of the mouse embryo [63]. However, to the best of our knowledge, there is no information regarding which particular tryptase is expressed in the mouse bladder and/or upregulated in the mouse model during inflammation. Although thrombin is a recognized physiological activator of PAR1 and PAR4, the endogenous enzymes responsible for activating PAR2 in urinary bladder are not known. Recently, it was demonstrated that the kallikrein family of proteinases are able to regulate PAR signaling and may represent important endogenous regulators PAR1, PAR2, and PAR4 [67]. The latter are likely to be confirmed in the urinary tract, since members of the kallikrein family play a fundamental role in bladder physiology [68]. It is interesting to note that activation of PARs on the bladder urothelium leads to activation of one of the members of the MiTF family. Others have shown that mast cell tryptase and MiTF are highly sensitive and specific markers for mast cell disease [69]. Moreover, MiTF regulates the expression of mouse tryptases, such as mcpt-7 [70] and mcpt-6, in response to stem cell factor [64] or transforming growth factor-beta stimulation [71].

### Involvement of mast cells and PARs in bladder inflammation

Mast cells are strategically located for optimal interaction with the environment and for their putative functions in host defense [72]. Among the mast cell mediators, tryptase is expressed by most human mast cells comprising as much as 25% of the cells' protein [73], and it is released in response to inflammatory stimuli [73,74]. Indirect evidence for a role of mast cells in cystitis was reviewed in Ref. [75] and includes increased concentration of tryptase in the urine of interstitial cystitis (IC) patients [9], presence of mast cells containing tryptase in the bladder of IC patients [75,76], and that mast cell counts in IC patients are one of the few features significantly associated with night-time frequency (p < 0.01; ref. [77]). We published direct evidence that mast cells are essential for both bladder inflammation and gene-regulation in experimental cystitis [7,8]. Our publications show that bladder inflammation is induced in normal mice and not in mast cell-deficient Kit^w^/Kit^w-v ^mice. The differences observed were abolished when Kit^w^/Kit^w-v ^mice had their mast cell deficiency selectively repaired by transplantation of bone marrow-derived mast cells from the normal congenic +/+ mice [7,8]. As the bladder response in these mice is similar to the normal +/+ mice, we concluded that mast cells are essential for the development of cystitis [7,8].

PARs provide an answer to the question of how mast cell products, such as tryptase, produce signals and induce bladder inflammation. Recently, we characterized the bladder responses to PAR-APs and presented evidence of a mandatory role for PARs in experimental cystitis [22]. We went further to determine the regulatory network downstream of PARs [25]. Our present findings further emphasize that products of mast cells such as tryptase inducing activation of PARs can lead to increased activity of transcription factors such as TFEB that play a fundamental role in mast cell survival. This positive feedback loop might be responsible for the mast cell-dependent perpetuation of bladder inflammation. However, the precise mechanisms involved in this feed back remain to be determined. One possibility is that the mast cells themselves express PARs and are susceptible to PAR-AP activation [78,79]. Indeed, PAR1-AP induces mast cell adhesion to fibronectin [79]. In contrast, messenger RNA for PAR-1 was detected in peritoneal mast cells [80], but PAR-APs failed to induce histamine release from these cells [80]. Another possibility is that inflammatory mediators can modulate the mast cell sensitivity to PAR-APs. Indeed, inflammatory cytokines such as IL-4 and IL-12 were suggested to regulate PAR receptor expression on the mast cells [81].

## Conclusion

To the best of our knowledge, this is the first report describing TFEB expression in response to PAR activation in the bladder and suggest a unifying, mechanistic pathway of bladder inflammation as follows: we hypothesize that mast cells release tryptase resulting in increased PAR activation and, consequently, TFEB/MiTF activity. As a result of increased of MiTF activity in mast cells there is mMCP-6 and mMCP-7 synthesis, leading to additional signals for inflammation. The findings that TFEB is downstream of activation of all PARs, makes this transcriptional factor a novel therapeutic target for the treatment of inflammatory disorders of the bladder.

## Abbreviations

CHIP, chromatin immunoprecipitation; IHC, immunohistochemistry; PAR, protease-activated receptors; PAR-AP, protease-activated receptor-activating peptide; Q-PCR = real-time polymerase chain; TF, transcription factor.

## Competing interests

The authors declare that they have no competing interests.

## Authors' contributions

All authors read and approved the final manuscript. **RS **conceived of the study and drafted the manuscript; **JM **participated on the design of immunohistochemistry protocols and choice of antibodies, performed the immunohistochemistry and confocal microscopy analysis; **BF **participated on the design of confocal experiments; **CAD **maintained the genotyping and animal colony; **CS **helped **MRS **with animal experiments, performed protein/DNA combo array, EMSAs, and gel shift assays, **ID **performed the statistical analysis of protein/DNA combo array, **MI **participated in its design and helped to draft the manuscript, **REH **participated in its design and helped to draft the manuscript, **BW **consulted **RS **regarding the participation of mast cells, **MRS **participated in its design, carried out all the animal experiments, and removed the tissues.
